# Development and Structural Analysis of Antibody Therapeutics for Filoviruses

**DOI:** 10.3390/pathogens11030374

**Published:** 2022-03-18

**Authors:** Xiaoying Yu, Erica Ollmann Saphire

**Affiliations:** 1 Center for Infectious Disease and Vaccine Discovery, La Jolla Institute for Immunology, 9420 Athena Circle, La Jolla, CA 92037, USA; xiaoyingyu@lji.org; 2 Department of Medicine, University of California, San Diego, La Jolla, CA 92093, USA

**Keywords:** antibody therapeutic, cryo-EM, ebolavirus, filovirus, marburgvirus, structural biology, X-ray crystallography

## Abstract

The filoviruses, including ebolaviruses and marburgviruses, are among the world’s deadliest pathogens. As the only surface-exposed protein on mature virions, their glycoprotein GP is the focus of current therapeutic monoclonal antibody discovery efforts. With recent technological developments, potent antibodies have been identified from immunized animals and human survivors of virus infections and have been characterized functionally and structurally. Structural insight into how the most successful antibodies target GP further guides vaccine development. Here we review the recent developments in the identification and characterization of neutralizing antibodies and cocktail immunotherapies.

## 1. Introduction

The Filoviruses belong to the family *Filoviridae* and are among the world’s deadliest pathogens. Among the six genera are the *Ebolaviruses*, the *Marburgviruses*, the *Cuevavirus*, the recently discovered *Dianlovi* rus [1], *Striavirus* [2], and the *Thamnovirus* [2]. There are 11 species in total, of which the ebolaviruses and marburgviruses are known to cause severe disease in humans. The genus *ebolavirus* includes six known viruses that are each antigenically distinct and named after the location of the disease outbreak where they were first identified. These include Ebola virus (EBOV), Sudan virus (SUDV), Bundibugyo virus (BDBV), Reston virus (RESTV), Taï Forest virus (TAFV), and Bombali virus (BOMV). The *Marburgvirus* genus contains Marburg virus (MARV) and its variant Ravn (RAVV).

The first filovirus to be identified, MARV, was discovered in 1967 when several laboratory workers in Germany developed hemorrhagic fever after handling tissues from non-human primates (NHPs). A total of 31 people were infected, and 7 died [3]. EBOV was first identified in 1976 when two separate outbreaks occurred in northern Zaire (now the Democratic Republic of Congo, DRC) and southern Sudan. Each outbreak resulted in hundreds of cases with 88% and 53% case fatality, respectively [4,5]. *Ebolavirus* has since appeared sporadically in Africa. The largest outbreak to date, which occurred in West Africa from 2014 to 2016, caused more than 28,600 infections and more than 11,300 deaths from Ebola Virus Disease (EVD). In 2021, two additional outbreaks occurred in the Democratic Republic of Congo [6] and Guinea [7]. Other filoviruses have similar outbreak potential and lethality. The most recent significant emergence of MARV in Angola had a case fatality rate of 90% [8]. Meanwhile, Sudan virus (SUDV) and the newly emergent Bundibugyo virus (BDBV) have case fatality rates of ~50% and 25–50% [9], respectively.

Symptoms of EVD include fever, headache, muscle pain, weakness, fatigue, diarrhea, vomiting, stomach pain, and hemorrhage (severe bleeding) [10,11]. The infection prodrome of filoviruses is virtually identical to common, co-circulating diseases like typhoid fever and malaria [10]. As such, early diagnosis, particularly for those cases early in a disease outbreak, is challenging. Given this potential delay in diagnosis, the therapeutic window for potential treatments must be broad so that treatments are effective even if delivered late in a disease course. Traditional approaches involving post-exposure vaccination and small molecule interventions required almost immediate administration to be effective [12]. Currently, monoclonal antibody (mAb) therapy has been shown to be the most effective route of therapy after symptoms appear, and can confer 100% protection for non-human primates (NHPs), even if administered as late as 5 days post-challenge [13,14]. Therefore, studies of antibodies against filoviruses are an important source for potential reliable therapeutics.

In recent years, progress has been made towards vaccine and treatment development. The first vaccine to be approved, Ervebo, is rVSV-based and was tested in an open-label, cluster-randomized ring vaccination trial in Guinea in 2015 [15], deployed in 2018 in the DRC under compassionate use, before gaining approval from the United States Food and Drug Administration (FDA) in late 2019 [16,17]. Another vaccine candidate utilizes a two-dose heterologous vaccination regimen with a replication-deficient adenovirus type 26 vector-based vaccine expressing a Zaire Ebola virus glycoprotein (Ad26.ZEBOV) and a modified vaccinia Ankara (MVA) vector-based vaccine, encoding glycoproteins from the Zaire EBOV, SUDV, and MARV as well as TAFV nucleoprotein (MVA-BN-Filo). This vaccine has been granted marketing authorization by the European Medicines Agency in the European Union [18,19].

The mAb monotherapy mAb114 and antibody cocktail REGN-EB3 were tested in clinical trials and proved to be effective against EBOV; both were granted FDA approval to treat EVD, and showed superior outcomes in reducing mortality compared to ZMapp and remdesivir [20,21,22]. The longer and multiple-dose regimen required for ZMapp and remdesivir administration could contribute to the slower rate of viral clearance of patients in those groups, and further lead to the difference in mortality between groups [20]. The intrinsic difference between the patient conditions among the four groups may contribute to the variation in treatment protection outcomes [20]. Notably, however, the therapeutic antibodies for humans approved thus far are only effective against EBOV. None show broad reactivity against other pathogenic filoviruses. Efficacious treatments against a range of pathogenic filoviruses are urgently needed.

## 2. Viral Entry and Glycoprotein Structure

**Figure 1:**
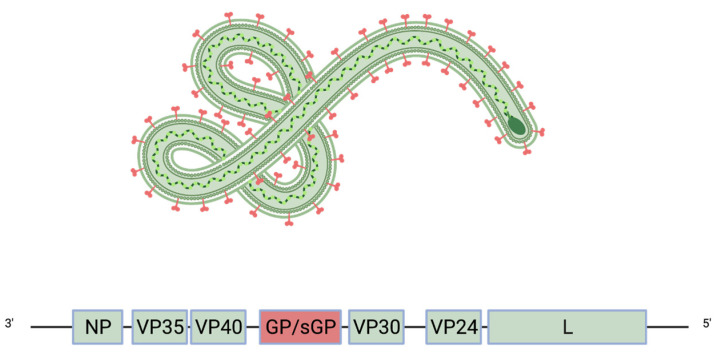
Schematic of the Ebola virus genome and virion. The glycoprotein GP (red) is the only viral protein displayed on the virion surface.

Filoviruses are enveloped, non-segmented, negative-sense RNA viruses that have a characteristic filamentous shape. The genome has seven genes that encode eight proteins (Figure 1). Six proteins are encoded by the corresponding viral genes, including VP24, NP, VP30, VP35, VP40 (matrix protein), and the RNA-dependent RNA polymerase (L). The ebolavirus *GP* gene expresses two major products: the trimeric glycoprotein, termed GP, which is displayed on the viral surface, and a dimeric, soluble version, termed sGP that represents the majority (80%) of the transcripts and is abundantly shed from infected cells [23,24,25,26]. GP and sGP share 295 amino acids and have some similarities in the folding of the monomeric unit [23,24,25,26]. The function of sGP remains unclear [23,27], but it has been proposed to act as an immune decoy [28]. Indeed, multiple antibodies cross-react with sGP and GP [29,30]. These antibodies may be absorbed by the much more abundant sGP and thus unavailable to neutralize virtual-surface GP. Many cross-reactive antibodies have a higher affinity for sGP [29,31], and its abundance indicates it may be a major antigen in a natural infection. Interestingly, marburgviruses and dianloviruses do not produce sGP; cuevaviruses express sGP similarly to ebolaviruses [31].

**Figure 2:**
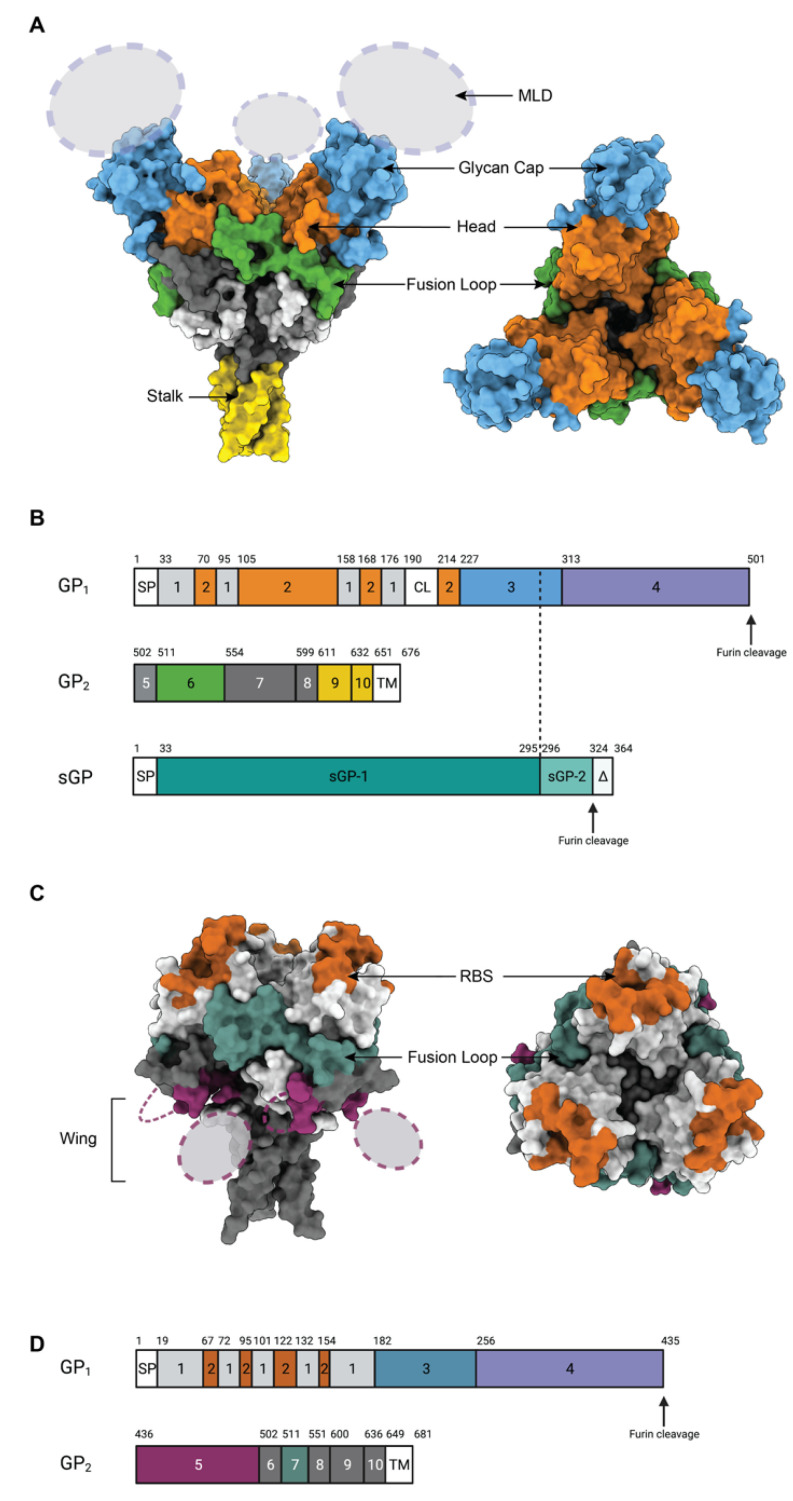
Epitopes on the GP surface. (**A**) Surface representation of Ebola Virus GP structure (PDB: 5JQ3) colored by domain. Side view and top view of Ebola virus GP are illustrated. (**B**). Schematic of the EBOV GP sequence. Amino acid numbering is at top, and polypeptide regions that form key domains are numbered in the center of the schematic blocks. 1: portions of the N-terminus of GP1 that form the base, 2: receptor-binding head, 3: glycan cap, 4: mucin-like domain (MLD), 5: GP2 N-terminal peptide; 6: fusion loop, 7: Heptad repeat 1 (HR1), 8 and 9: Heptad repeat 2 (HR2); 9: stalk; and 10: and membrane-proximal external region (MPER), respectively. Other regions include SP: signal peptide, and TM: transmembrane domain. The organization of sGP is illustrated below. The first 295 residues are identical to those in GP1 (labelled sGP-1). Residues 296 through 324 are unique to sGP (labelled sGP-2). The C-terminal sequence, termed delta peptide, is released from sGP by furin cleavage. (**C**) The surface representation of Marburg Virus GP structure (PDB: 6BP2) colored by domain. Side view and top view of Marburg virus GP are illustrated. (**D**) Schematic of the MARV GP sequence. Amino acid numbering is at top. 1–2: GP1, with 2 for RBS; 3: glycan cap, 4: MLD, 5: wing; 6: N-terminal loop: 7: fusion loop, 8: HR1, 9: HR2; 10: MPER; SP: signal peptide, and TM: transmembrane domain.

GP mediates attachment and entry into target cells. As the only surface-exposed protein on mature virions, GP is the focus of current filovirus therapeutic mAb discovery efforts (Figure 2) [32]. Filovirus GPs share a common core fold and trimeric organization, but are antigenically distinct due to species-specific sequence differences of up to 70%. The ebolavirus GP monomer comprises GP1 and GP2 subunits that are anchored together by a single GP1-GP2 disulfide bond [33]. The larger GP1 subunit harbors the receptor-binding site (RBS), the glycan cap domain, and the heavily glycosylated mucin-like domain (MLD). GP2 contains the membrane fusion machinery, including the internal fusion loop (IFL), two heptad repeats (HR1 and HR2), the membrane-proximal external region (MPER), and the transmembrane domain (TM) [25]. The GP2 subunit, particularly the IFL and stalk regions, has greater sequence conservation than GP1 among filoviruses. Marburgvirus GPs have a similar arrangement (Figure 2C,D). However, in marburgvirus GP, the furin cleavage site is shifted towards the N-terminus (residue 435 for Marburgvirus vs. 501 for ebolavirus), the region corresponding to the MLD is split into two halves: the major portion of the MLD is attached to the C terminus of GP1. The minor portion (residues 436–501) is attached to the N terminus of the GP2 and is termed the wing domain [34,35].

Filoviruses enter cells via macropinocytosis [36,37,38]. Once in the endosome, GP is cleaved by host endosomal cathepsins that remove both the MLD and the glycan cap [39,40,41] to form cleaved GP (GP_CL_). In GP_CL_, the RBS for the cellular receptor Niemann-Pick C1 (NPC1) is exposed at the apex of GP1 [42]. Following receptor binding, the fusion subunit in GP_CL_ rearranges into a six-helix bundle that mediates fusion between host and virus membranes [43].

## 3. Efforts for Antibody Discovery

Analyses of antibody responses in human survivors of virus infection can outline key characteristics of antibodies elicited in response to infection and provide an important basis for the development of therapeutic antibodies.

Beginning in the early 1990s, isolation of neutralizing mAbs was mostly enabled by display library approaches, such as phage display [44,45]. Early antibodies like KZ52 and others were discovered from human survivors of the 1995 Kikwit outbreak in DRC [46]. These antibodies facilitated and enhanced understanding of the virus and permitted the determination of the structure of EBOV GP in its pre-fusion conformation [25]. Screening of antibody-secreting hybridomas from immunized mice produced a panel of novel antibodies that could be categorized into multiple different epitope groups [47]. This classification guided the formation of several successful mAb cocktails, including MB-003, ZMAb, and ZMapp, which had non-overlapping binding sites and high efficacy in NHP studies of EBOV infection [13,48,49].

Technological advancements, such as single B cell isolation and next-generation sequencing, accelerated large-scale mAb discovery, direct functional analysis, and exploration of the Ab maturation process. Potent antibodies were subsequently discovered in immunized animals [50,51,52], human survivors [30,53,54,55], and vaccinated human volunteers in clinical trials [56,57]. Notably, antibodies in these panels target a spectrum of epitopes on GP, and several neutralize broadly are active against several filovirus species. Several therapeutic antibody cocktails, including REGN-EB3, FVM04/CA45, MBP134^AF^, rEBOV-520/548, rEBOV-442/515, and 1C3/1C11, were generated and shown to be highly effective [52,58,59,60,61,62].

The functional activity of antibodies isolated during discovery efforts can be evaluated by in vitro neutralization assays to determine whether they block infection by one or more ebolaviruses together with structural biology to reveal the molecular basis for protective activity. Neutralization can be analyzed using authentic virus under BSL-4 containment [63] or at lower biosafety levels using model systems. The biologically contained Ebola virus ΔVP30 system can be performed at BSL-3 [64]. In this system, the entire open reading frame of VP30, which is an essential transcription factor for EBOV replication, is deleted to generate a replication-deficient particle. The VP30 needed for replication is supplied in *trans* through Vero cells that stably express VP30 such that EBOV∆VP30 can undergo multiple replication cycles only in VeroVP30 cells, but not the parental cells that lack VP30. In this system, all viral antigens and proteins, including sGP, are stably expressed. A second neutralization system involves recombinant vesicular stomatitis virus (VSV) bearing EBOV GP [65] and can be performed at BSL-2. This method uses a recombinant VSV (rVSV) in which the VSV-G gene is replaced with filovirus GP that is then displayed on the rhabdoviral surface [65,66,67,68]. These pseudovirus constructs typically carry a fluorescence reporter such as GFP to monitor infection, and are frequently modified so that only GP, and not secreted sGP, is expressed.

A systematic analysis of 171 mAbs by The Viral Hemorrhagic Fever Immunotherapeutics Consortium (VIC) compared the readouts of 3 different neutralization assays by epitope and level of in vivo protection [31]. There were several differences in the performance of the 171 mAbs across the 3 assays. For example, the authentic EBOV assay was more forgiving: a group of glycan-cap-directed antibodies only neutralized authentic EBOV and no model system. The ∆VP30 system was more stringent: fewer antibodies demonstrated neutralization overall. The results that correlated best with in vivo protection, however, were those assays that contained sGP (i.e., authentic EBOV and ∆VP30), as well as the fraction left un-neutralized, which was measured in rVSV assay. Antibodies that failed to completely neutralize rVSV at the highest concentration similarly failed to protect in the mouse model.

A small fraction of antibodies had no neutralization activity in any assay, yet nevertheless protected in a mouse model of EBOV infection. In high-throughput systems-serology assays, which examined the contribution of immune effector functions like phagocytosis and activation of natural killer (NK) cells to in vivo antibody efficacy, these antibodies had high immune effector function activity, indicating the potential contribution of Fc-mediated activity to protection [31,69]. By comparison of the in vivo activity of the Fc variants of mAbs from different epitope groups, a previous study revealed the differential requirements for FcγR to achieve protection [70]. MAbs targeting the membrane-proximal regions (HR2 and MPER) or the MLD do not inspire Fc–FcγR interactions. However, the mAbs that contact to chalice bowl region or the fusion loop are linked to FcγR engagement. Moreover, by applying an Fc engineering platform, a library of Fc variants of a specific mAb could be generated to compare how Fc effector functions correlate with mAb protection performance. Based on the results of functional characterization of the variants and the in vivo protection in mice, Fc variants with high complement activity, yet moderate and balanced ADCC activity are more protective against viral infection in vivo [71]. Together, the previous results suggested that effective antibody treatments should comprise mAbs that achieve both neutralization and immune effector functions. The VIC study and other work [31,51,53,54] also revealed the epitopes at which broader ebolavirus cross-reactivity can be achieved.

Structural biology has served a vital role in increasing our understanding of the molecular mechanisms underlying antibody-mediated neutralization of many viral infections. In particular, atomic resolution structures obtained using cryogenic electron microscopy (cryo-EM) or X-ray crystallography allow exploration of the fine details of binding between antibodies and virus proteins like GP to reveal crucial information about molecular interactions and the basis of cross-reactivity. Combining structural studies with virology and in vitro biochemistry allows a thorough evaluation of therapeutic candidates and their target interactions.

### 3.1. Structural Biology to Reveal Epitopes on GP Targeted by Antibodies

Antibodies isolated from immunized animals or patients infected with ebolavirus have been shown to target several different regions on the surface of the GP trimer. Epitope mapping can be achieved rapidly using competition binding assays or negative stain electron microscopy (EM). However, to definitively understand the antibody interactions with glycoprotein, a high-resolution structure by X-ray crystallography or cryo-EM is required. Structural characterizations of these antibodies in complex with full-length GP ectodomain or GP peptides provide detailed information of the neutralizing mechanism and inform new approaches for broad immunotherapy and vaccine design. The recent development of single-particle cryo-EM capabilities facilitated the determination of more structures of antibody-glycoprotein complexes, including those that involve asymmetric interactions that are difficult to assess by crystallography. As a whole, the structural analyses illustrate how antibodies target the various epitopes on the GP surface, particularly those epitopes in highly conserved regions, to achieve high potency and/or cross-reactivity.

### 3.2. mAbs Targeting the Glycan Cap

In EBOV, the glycan cap spans between residues 227 and 312, and the majority of residues (amino acids 227–295) are present in both GP and sGP (Figure 2). Thus, antibodies targeting the GP glycan cap typically also react with the abundant, non-structural sGP. If these antibodies were elicited by natural infection, they may, in fact, have been elicited against sGP, which is at least five-fold more abundant than membrane-bound GP. One component of the therapeutic cocktail ZMapp [13,72,73], 13C6 [74], targets the glycan cap and offers protection in in vivo models of infection despite having low neutralizing potency [29,72].

**Figure 3:**
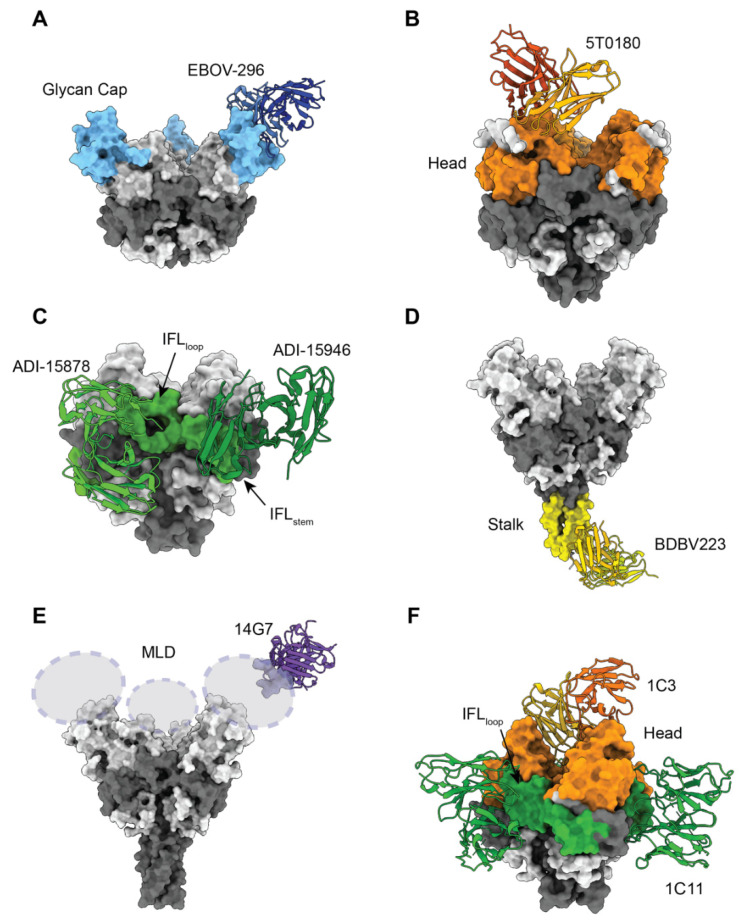
Structural models of neutralizing antibody recognition against key epitopes on the GP surface. GP epitopes are colored as in Figure 2. The variable regions of the antibodies targeting different epitopes are shown in cartoon representation. (**A**) Glycan cap-targeting antibody EBOV-296 (PDB: 7KF9) (**B**) Head-region targeting antibody 5T0180 (PDB:6S8J) (**C**) IFL-targeting antibody ADI-15878 and ADI-15946 (PDB: 6EA5, 6MAM) (**D**) Stalk region-targeting antibody BDBV 223 (PDB: 6N7J, 5JQ3) (**E**) MLD-targeting antibody 14G7 (PDB: 2Y6S. 5JQ3) (**F**) An example of a broad neutralizing antibody cocktail 1C3 and 1C11, with 2 antibodies targeting the head and IFL, respectively. (PDB: 7SWD).

Antibodies against the glycan cap, including 13C6, can be characterized by higher levels of immune effector functions [69]. Some neutralize as well, and several have been characterized functionally and structurally, such as EBOV-548 and EBOV-296 (Figure 3A) [60,75]. Approaching the glycan cap via different angles, anti-glycan cap mAbs with GP largely involve CDRH3 or CDRH2 that mimic and displace the β18-18′ hairpin, which acts as an anchor for the MLD. The proposed mechanism of neutralization for these anti-glycan cap antibodies is blockage of the cathepsin cleavage event that is required for RBS exposure and viral entry [75]. Higher numbers of contacts between a mAb and the MLD cradle are shown to introduce instability in the GP trimer, and thus these antibodies can synergize with those that target the fusion loop. Other glycan-cap targeting mAbs include the Q206, Q314, and Q411 antibodies identified in immunized macaques, which provide partial protection in a mouse model of EBOV challenge [76]. Overall, mAbs in this group are usually potent but rarely have broad neutralizing activity. However, a combination of both neutralizing and effector functions and the demonstrated synergistic effect when pairing with mAbs that target the fusion loop, make some of the more potent glycan cap mAbs good candidates for inclusion in therapeutic cocktails [54,60,75].

### 3.3. mAbs Targeting the Apex/Head/Receptor Binding Region of GP

The GP1 Head epitope lies under the glycan cap and contains residues that are part of the RBS. In the late endosome, primed GP_CL_ exhibits a fully exposed RBS that is competent for binding to domain C of NPC1 (NPC1-C) [42]. In contrast to ebolaviruses, the glycan cap of marburgviruses provides a less complete shield and the RBS is more exposed prior to cleavage, such that several antibodies including MR78 and MR191 can target the RBS directly [77,78,79]. MR78 and MR191 somewhat mimic interactions made by a loop of NPC1-C, which contains aromatic residues that can reach into a hydrophobic cavity on GP_CL_ [42].

The monotherapy mAb114, which was isolated from a survivor of the 1995 Kikwit EVD outbreak, and is approved to treat EVD, targets the head epitope in the RBS via a near-vertical angle to block receptor binding [80]. FVM04, isolated from immunized macaques [50], binds to the inner chalice of the GP trimer near the glycan cap with a tilted angle, so that from low-resolution negative stain EM, only one Fab bound to GP trimer can be visualized [81]. FVM04 can bind and neutralize both EBOV and SUDV but has lower activity toward BDBV [81]. Other head-targeting antibodies (5T0180, 1T0227, and 3T0265), isolated from rVSV-ZEBOV vaccine recipients, also bind the RBS and block NPC1-C, while avoiding contact with the glycan cap region [82] (Figure 3B). Although the residues within the footprint of these 3 antibodies are mostly conserved, a key difference between EBOV and BDBV/SUDV at residue 224 (G in EBOV, N in BDBV/SUDV) sterically prevents their binding to BDBV/SUDV GP, thus limiting the breadth of antibody potency [82]. Overall, previously discovered head targeting neutralizing antibodies are potently neutralizing and protective, but limited in breadth.

A recently published apex-targeting antibody 1C3 is unique among currently characterized EBOV antibodies in that it targets the center of the GP chalice and binds one Fab to one GP trimer (Figure 3F) [62]. 1C3 potently neutralizes both EBOV and SUDV. Although 1C3 does not neutralize BDBV, it does bind to recombinant BDBV GP in ELISA, suggesting that this antibody could potentially contribute to protection against BDBV infection through Fc-dependent effector functions [62]. The asymmetric binding with 1:1 stoichiometry of 1C3 allows more variability within its footprint [83]. Notably, the quaternary recognition of 1C3 is specific for GP and not shed sGP, which may provide an advantage for mAb therapeutic candidates.

### 3.4. mAbs Targeting Internal Fusion Loop (IFL)

The IFL region (residues 511–554) plays a critical role in viral-host membrane fusion. This important role translates to a high degree of sequence conservation (60–70%), which makes the IFL an ideal epitope for cross-reactive antibodies. The IFL region can be further divided into stem/base (IFL_stem_; residues 511–520 and 543–554) and the loop/paddle (IFL_loop_; residues 521–542) [84]. MAbs targeting the IFL usually also contact parts of the base epitope, which is immediately adjacent to the IFL and forms the base of the “bowl” of the GP chalice. The central span of GP, including both the IFL and base, has been termed the “waist” and is targeted by antibodies via a continuum of antibody epitopes [85]. Several cross-reactive antibodies identified thus far have been categorized into the IFL targeting group, including CA45 [86,87], 6D6 [88], ADI-15946 [89], ADI-15878 [85], 2G1 [90], EBOV-520 [60], EBOV-515 [61], and 1C3 [62]. Among these mAbs, 6D6, ADI-15878, and 1C3 have similar footprints that overlap and include the IFL_loop_ region and part of the base region (although the 6D6 complex structure has been resolved only at low-resolution with negative stain EM). Meanwhile, CA45, ADI-15946, EBOV-520, and EBOV-515 have similar footprints that include both the IFL_stem_ and other parts of the base region (Figure 3C). 2G1, isolated from a vaccinated donor and which cross-neutralizes pseudotyped EBOV, SUDV, and BDBV, was determined to bind GP2 by competition assays and was further mapped to the fusion loop by computational modeling [90].

ADI-15878 contacts the IFL_loop_ and the portion of the base termed the N-terminal pocket, which is occupied by the flexible N-terminal tail of GP2 in the GP apo-structure. The residues that line the N-terminal pocket are highly conserved, but those in the flexible N-terminal tail are not. Therefore, the ability of the ADI-15878 CDRs to reach the conserved pocket region underneath the N-terminal tail provides the cross-reactivity against different ebolaviruses [85]. CA45 targets both the IFL_stem_ and a region termed the DFF cavity [87,91], which is occupied by a short flexible loop that includes residues 192–194 near the cathepsin cleavage site (residues DFF) in the apo GP structure. The footprint of 1C11 partially overlaps that of ADI-15878, but is shifted upwards [62].

ADI-15946, EBOV-520, and EBOV-515 all contact the IFL_stem_ and a region termed the 3_10_ pocket, the core of which encompasses residues 71–75 of GP1. The pocket extends to surrounding residues, including 76–78 of GP1 and 510–516 of GP2 [60,89]. This region is occupied by the β17-β18 loop (residues 287–291), which is part of the glycan cap in the uncleaved GP apo-structure and is exposed in GP_CL_ following removal of the glycan cap. Similar to the crystal structure of the ADI-15946-GP_CL_ complex, the cryo-EM structure of EBOV-520 in complex with uncleaved GP ectodomain shows that the CDRH3 loop of the antibody contacts the 3_10_ pocket [60]. Although the glycan cap region is intact in the EBOV-520 complex structure, the β17-β18 loop cannot be visualized, suggesting that this flexible loop is displaced upon contact with the mAb [60].

ADI-15946 potently neutralizes EBOV and BDBV, but not SUDV, whereas EBOV-520 neutralizes all three viruses. Comparing the footprints of the two mAbs, EBOV-520 is shifted slightly upward to avoid non-conserved position 506 (N506 in EBOV and R506 in SUDV), which may allow the extra reactivity towards SUDV GP [60]. The study on ADI-15946 also provided a good example of structure-based rational engineering. Three residues were substituted to reduce steric and charge clashes with the non-conserved residues in SUDV (R100A in CDRH3) or to improve binding by generating a double tyrosine binding motif (S65Y and F67Y in the FRL3). The designed mAb variant successfully expanded the breadth of ADI-15946 to enhance its binding and neutralization against SUDV [89].

Overall, mAbs that target the IFL region enlist a variety of approaches to contact the most conserved region on the GP surface and showcase the largest number of broadly neutralizing antibodies that have been discovered and characterized to date. The identification of the flexible loops in GP that potentially compete with the mAbs from this group suggests that removal of such regions would contribute to improved antigens and facilitate the development of more antibodies that target these ideal epitopes.

### 3.5. mAbs Targeting GP Stalk and MPER Region

The stalk/MPER region lies near the C terminus of GP2 above the transmembrane domain. As part of the fusion machinery, the stalk/MPER region has high sequence conservation across filovirus species and is a prime target for mAbs that have broad potency. Negative stain EM was used to map the binding footprint of several cross-reactive antibodies that target the stalk region (BDBV 223, BDBV 317, BDBV 340, and ADI-16061) [54,55]. A high-resolution X-ray crystal structure of the Fab from BDBV 223, isolated from a survivor of BDBV infection, was determined in complex with a synthetic peptide of the epitope region was determined [92] (Figure 3D). Interestingly, the alignment of the complex structure to the GP trimer structure and tomographic reconstruction of the GP trimer on the virus membrane [93] revealed that BDBV 223 binding interferes with the trimeric bundle assembly and anchoring of the GP spike in the viral membrane. Thus, interference with the six-helix bundle formation needed to drive membrane fusion could be a key mechanism by which BDBV 223 neutralizes infection [92].

### 3.6. mAbs Targeting Mucin-Like Domain

The mucin-like domain (MLD) is a heavily glycosylated region located on the C-terminus of GP1 that shields the top of the GP trimer. The MLD has the most sequence variation among various species. Due to its highly flexible nature, the structure of MLD is not well characterized, and thus the mAbs targeting MLD are less understood. The residues required for binding of MLD mAbs were identified by peptide binding assays [47] or by alanine scanning, which evaluates how mutations at individual residues affect binding to EBOV GP [74]. Several mAbs targeting EBOV MLD have been discovered, including the MB-003 cocktail mAbs 6D8 and 13F6, which exhibit low or no neutralization in vitro [47], yet protected in the mouse challenge model, and, collectively with 13C6, provide protection in the NHP model [49]. Efforts have been made to co-crystalize mAb Fab fragments in complex with peptides of identified epitopes. Such structures have been determined for 13F6 [94] and another mAb targeting the MLD, 14G7 [95] (Figure 3E).

In marburgviruses, the unique wing domain located at the N terminus of GP2 is also part of the MLD. Although this domain has not been fully characterized structurally and functionally, four mAbs targeting this region have shown 90–100% protection in the mouse challenge model [35]. Studies on two wing-specific antibodies, MR228 and MR235, identified from human survivors revealed more features of mAbs in this epitope group. MR228 is non-neutralizing but protective in the mouse model, and its protective activity is likely mediated by Fc effector functions, specifically the engagement of FcγRs [96]. MR235 does not protect in in vivo models of infections, yet cooperatively enhances binding of RBS-targeting neutralizing antibodies by facilitating the structural rearrangement of marburgvirus GP [96]. Overall, mAbs targeting the MLD are less likely to be neutralizing [31] but may offer protection through Fc-mediated functions [74,96].

### 3.7. mAb Cocktail Immunotherapies

For more than a decade, studies exploring mAbs against filoviruses have demonstrated the potential of using a single or a cocktail of mAbs as immunotherapy, leading to two approved mAb treatments, mAb114 and the cocktail REGN-EB3 in 2020. However, the uncertainty of the causative agents of the next viral outbreak requires a versatile toolbox. The usage of high quantities of mAbs to treat disease caused by filovirus infection presents challenges in production and in cost. Therefore, the development of mAb cocktails as immunotherapies aims to achieve broader reactivity and lower dosage.

First-generation cocktail immunotherapies such as MB-003, ZMAb, and ZMapp can protect against EBOV challenge in NHP [13,48,49]. MB-003 contains antibodies against the MLD and the glycan cap, whereas ZMAb is composed of antibodies against the glycan cap and the base domain. ZMapp is derived from both MB-003 and ZMAb, and combines one mAb from MB-003 with two mAbs from ZMAb, with one mAb (13C6) against the glycan cap and two against the base (2G4 and 4G7) [72,74]. ZMapp was the first antibody cocktail shown to reverse severe disease in the NHP model [13]. During the 2014–2016 outbreak in West Africa, the ZMapp and ZMAb cocktails were used to treat 25 EVD patients under compassionate use protocols in several countries [97]. However, the benefits of the cocktail therapeutics themselves could not be determined definitively since these patients also received other aggressive supportive measures [98]. In a randomized controlled clinical trial, administration of ZMapp was beneficial against human EVD but did not meet the efficacy threshold compared to patients who received the current standard of care alone [99].

REGN-EB3 is a second-generation cocktail of three mAbs, REGN3470, REGN3471, and REGN3479, each isolated from Velocimmune mice, which have human immunoglobulin variable regions [52]. REGN3470 targets the glycan cap from the side, with a binding angle parallel to the viral surface. REGN3471 targets the GP1 head region at the inner chalice, with an angle perpendicular to the viral surface, and REGN3479 targets the fusion loop [52]. REGN-EB3 was superior to ZMapp in reducing EVD mortality in a randomized clinical trial [20], and was approved by the FDA in 2020. An antibody against the head domain, mAb114 [14], was similarly effective as a monotherapy [20]. A two-antibody cocktail including rEBOV-520 that targets the fusion loop region/base area, and rEBOV-548, which targets the glycan cap, is also effective in protecting NHP from EBOV infection [60].

With the uncertainty of viral species responsible for the next outbreak, the next generation of immunotherapy ideally will offer a cross-protective cocktail. In recent years, several mAb cocktails have been characterized and investigated in NHPs to demonstrate protection against viral infection. Two broadly neutralizing mAbs, FVM04 and CA45, have been evaluated as a cocktail in NHPs with EBOV and SUDV infections, and proved to be protective [58]. When supplemented with MR191, an anti-MARV mAb, the triple mAb cocktail exhibited full protection against death in MARV-infected NHPs [58]. In addition, antibody cocktail RIID F6-H2 is comprised of two SUDV specific mAbs, 16F6 and X10H2, targeting the base and glycan cap of GP, respectively [100,101]. This mAb cocktail protects macaques from the SUDV challenge with two doses on day 4 and day 6, at 25 mg/kg per mAb. The model is not fully lethal; 50% of the mock-treated exposed control animals survived the SUDV challenge [100].

A second cocktail named MBP134^AF^ contains two non-competing IFL targeting mAbs, ADI-15878 (described in IFL region mAb section) and ADI-23774, which was selected after specificity maturation of ADI-15946 to bind SUDV GP using yeast-display technology [102,103]. The mAb pair was further optimized to improve their capacity to activate NK cell functions by adopting all afucosylated glycans (thus the AF in the cocktail name), in order to reach higher efficacy against EBOV [31,102]. The cocktail protects NHPs against EBOV, SUDV, and BDBV [59].

A third cocktail, which also comprises two mAbs, rEBOV-442 and rEBOV-515, was recently reported to protect NHP from disease caused by EBOV, BDBV, and SUDV [61]. These two mAbs exhibited synergy in neutralization by occupying non-overlapping epitopes, with rEBOV-442 targeting the glycan cap region [75], and rEBOV-515 targeting the conserved IFL region. Compared to the previously described ADI-15946 [89] and EBOV-520 [60], the footprint of rEBOV-515 is more conserved and thus provides better neutralizing breath against SUDV [61].

The fourth cocktail of two human survivor antibodies was recently described [62]. This cocktail includes antibodies isolated from survivors of EVD: 1C3 and 1C11, and also protects NHP against lethal challenge with EBOV or SUDV [62]. The 1C3 and 1C11 pair was chosen from a broad analysis of the VIC consortium, and has been tested in multiple animal models (mouse, guinea pig, and NHP). 1C3 uniquely targets the head region with one Fab anchoring into the GP chalice to bind all the three monomers of the GP trimer simultaneously (Figure 3F). This tripartite recognition mode leads to strong binding to the GP trimer, and no cross-reactivity to the dimeric shed sGP. The GP specificity of 1C3 is unique for an EBOV GP head-binding antibody and results from its particular quaternary epitope recognition. Interestingly, different parts of 1C3 target the identical GP residues on each monomer. For example, GP residues D117 in monomer A forms hydrogen bonds with CDRH3 of 1C3, in monomer B forms hydrogen bonds to FRL3, and contacts FRL1 in monomer C [62]. The broadly reactive 1C11 antibody targets the fusion loop/base region via an epitope similar to that of 6D6 [88] and ADI-15878 [85]. 1C11 binds with three copies of the Fab per GP trimer, with each individual Fab bridging two adjacent monomers together to link the fusion loop paddle of monomer A to the neighboring monomer B, including the N-linked glycan at position 563 at each of the three positions around the trimer.

These third-generation candidate therapeutic cocktails can all protect NHP against infection by multiple ebolaviruses, representing the direction of therapeutic development in the field.

For the two approved EVD treatments, the mAb114 monotherapy required a 50 mg/kg dose, whereas the REGN-EB3 triple cocktail required 50 mg/kg of each mAb component for a total 150 mg/kg dose [21,22]. Here we also compare the recently reported cocktails that are protective in NHPs. FVM04/CA45 was protective against EBOV when offered at 40 mg/kg (20 mg/kg each) on day 4, and protective against SUDV when offered at 40 mg/kg (20 mg/kg each) on day 4, plus a second dose at 13 mg/kg (8 mg/kg FVM04, 5 mg/kg CA45) on day 6. Against MARV, MR191 was administered in addition to the two-mAb FVM04/CA45 cocktail at 50 mg/kg, with the first dose (90 mg/kg total) on day 4 and the second dose on day 6 (70 mg/kg total). The second cocktail, MBP134^AF^, was tested in NHPs against EBOV, SUDV, and BDBV, and showed protection with a single 25 mg/kg dose. However, in both studies, the mock-treated exposed control animals survived the SUDV challenge (one out of two in the FVM04/CA45 study, two out of four in the MBP134^AF^ study), which limits the significance of the protection results. In the MBP134^AF^ BDBV protection study, five out of six treated animals survived. The third cocktail, rEBOV-442/515, was tested in NHPs against EBOV, SUDV, and BDBV, and showed protection with a two-dose regimen at 30 mg/kg (10 mg/kg rEBOV-442, 20 mg/kg rEBOV-515). The fourth cocktail, 1C3/1C11, was protective against EBOV at 25 mg/kg, and protective against SUDV at 50 mg/kg, both with two doses on day 4 and day 7. The synergistic effect between mAbs described in several studies also hinted at the possibility that these dosages could be further optimized.

One limitation shared by these NHP studies is the relatively small number of animals per group, which results in lower statistical power for examination of the significance of the beneficial effect. Future studies will need to include additional NHPs to test the reported regimens and dosages and to explore the efficacy of single and lower dosages of the proposed cocktails.

## 4. Conclusions

After the neutralizing monoclonal antibody KZ52 was found not to protect NHPs infected with Ebola, it was initially thought to be an indication that mAbs may not be effective against rapidly progressing EVD [104]. The discovery that the three non-neutralizing antibodies of MB-003 could protect primates, and subsequent refinement and improvement of antibody cocktails to include neutralizing antibodies that targeted epitopes in the GP base allowed not only survival, but reversion of advanced disease symptoms, as demonstrated by ZMapp in NHPs [13], set a starting point for use of mAbs as therapeutics. The broad collaborative analysis of the VIC illuminated multiple antibody features that led to protection and proposed that antibody therapies ideally should offer potent neutralization, a lack of an un-neutralized viral fraction, and recruitment of Fc effector functions. Further, the analysis by the VIC indicated that the Fc effector function recruitment, particularly phagocytosis, could be strongest at the top of the molecule (e.g., head, glycan cap, and MLD), and that the head epitope, in particular, was a sweet spot that permits both effector function recruitment, as well as potent neutralization by blocking receptor binding [31]. The VIC work established standards, enabled cross-comparison, and allowed side-by-side evaluation of all the cocktail components thus far, including the second-generation mAb cocktails (MB-003, ZMAb, ZMapp, MBP134^AF^, REGN-EB3), and the proposed mAb cocktail (mAb 1C3 and 1C11) for the combination of complementary activities with antibodies of broad specificity.

Second-generation antibody treatments combine both neutralization and Fc functions, either by binding a single monotherapy at the head sweet spot (i.e., mAb114 [14]), or by combining antibodies having different epitopes and functions, as in REGN-EB3 [20]. The third generation of antibody treatments aims to confer protection against other disease-causing ebolaviruses, and ideally, involve a lower dosage or a simpler therapeutic regimen. These cocktails, including MBP134^AF^, rEBOV-520/548, rEBOV-442/515, and 1C3/1C11, have reduced the three-antibody cocktail to two, and each contains at least one mAb targeting the fusion loop region, with the other mAb binding to IFL, glycan cap, or the apex/head region [59,60,61,62]. Mapping the structures and activities of antibodies effective against different ebolaviruses illuminates not only emergency post-exposure treatment options, but also illustrates the types of antibodies that vaccines should elicit.

The role of sGP in the immune system during viral infection still remains largely unclear. In the VIC systematic review, sGP cross-reactivity did not significantly affect in vivo protection [31]. The approved monotherapy mAb114 cross-reacts with sGP, and protects against EVD in NHP studies and in the clinical trial at a 50 mg/kg dosage. However, the currently lowest treatment dosage to protect NHP, 25 mg/kg, was achieved by mAb cocktails MBP134^AF^ and 1C3/1C11, the components of which are specific to the GP trimer and do not bind to sGP [59,62]. This result could suggest that a lower effective dosage could be achieved using GP-specific antibodies alone. More studies are needed to better understand whether sGP is an immunogen or a decoy, so that we can address whether cross-reactivity against sGP is an advantageous feature or should be avoided for mAb candidates.

With the current vaccine only available for EBOV, there remains the need for the continued availability of antibodies as it is impractical to vaccinate all, and the frequency and unpredictable timing and location of outbreaks suggest continued treatment development is needed. Studies focusing on characterizing vaccine-elicited mAbs and comparison to those elicited from viral infection would also contribute to the next stage of broadly effective vaccine and immunotherapy development [56,57].

Lastly, platforms and expertise honed on the Ebola virus and the collaborative framework of the VIC [31] were both deployed in rapid development, advancement, and comparison of antibody therapeutics against SARS-CoV-2 [105], and will likely be called upon again against future emerging infections.

## Data Availability

Not applicable.
